# Long-term prognostic implications of brachial-ankle pulse wave velocity in patients undergoing percutaneous coronary intervention

**DOI:** 10.3389/fmed.2024.1384981

**Published:** 2024-06-07

**Authors:** Byung Sik Kim, Jong-Hwa Ahn, Jeong-Hun Shin, Min Gyu Kang, Kye-Hwan Kim, Jae Seok Bae, Yun Ho Cho, Jin-Sin Koh, Yongwhi Park, Seok-Jae Hwang, Udaya S. Tantry, Paul A. Gurbel, Jin-Yong Hwang, Young-Hoon Jeong

**Affiliations:** ^1^Division of Cardiology, Department of Internal Medicine, Hanyang University College of Medicine, Hanyang University Guri Hospital, Guri-si, Republic of Korea; ^2^Department of Internal Medicine, Gyeongsang National University School of Medicine and Cardiovascular Center, Gyeongsang National University Changwon Hospital, Changwon-si, Republic of Korea; ^3^Department of Internal Medicine, Gyeongsang National University School of Medicine and Division of Cardiology, Gyeongsang National University Hospital, Jinju-si, Republic of Korea; ^4^Sinai Center for Thrombosis Research and Drug Development, Sinai Hospital of Baltimore, Baltimore, MD, United States; ^5^CAU Thrombosis and Biomarker Center, Chung-Ang University Gwangmyeong Hospital, Gwangmyeong-si, Republic of Korea; ^6^Division of Cardiology, Department of Internal Medicine, College of Medicine, Chung-Ang University, Seoul, Republic of Korea

**Keywords:** coronary artery disease, arterial stiffness, pulse wave velocity, percutaneous coronary intervention, clinical outcome

## Abstract

**Objective:**

The long-term clinical effect of arterial stiffness in high-risk disease entities remains unclear. The prognostic implications of brachial-ankle pulse wave velocity (baPWV) were assessed using a real-world registry that included patients who underwent percutaneous coronary intervention (PCI).

**Methods:**

Arterial stiffness was measured using baPWV before discharge. The primary outcome was net adverse clinical events (NACE), defined as a composite of all-cause death, non-fatal myocardial infarction, non-fatal stroke, or major bleeding. Secondary outcomes included major adverse cardiac and cerebrovascular events (MACCE: a composite of all-cause death, non-fatal myocardial infarction, or non-fatal stroke), and major bleeding. The outcomes were assessed over a 4-year period.

**Results:**

Patients (*n* = 3,930) were stratified into high- and low-baPWV groups based on a baPWV cut-off of 1891 cm/s determined through time-dependent receiver operating characteristic curve analysis. baPWV was linearly correlated with 4-year post-PCI clinical events. The high baPWV group had a greater cumulative incidence of NACE, MACCE, and major bleeding. According to multivariable analysis, the high baPWV groups had a significantly greater risk of 4-year NACE (adjusted hazard ratio [HRadj]: 1.44; 95% confidence interval [CI]: 1.12–1.85; *p* = 0.004), MACCE (HRadj: 1.40; 95% CI: 1.07–1.83; *p* = 0.015), and major bleeding (HRadj: 1.94; 95% CI: 1.15–3.25; *p* = 0.012).

**Conclusion:**

In PCI-treated patients, baPWV was significantly associated with long-term clinical outcomes, including ischemic and bleeding events, indicating its value for identifying high-risk phenotypes.

## 1 Introduction

Coronary artery disease (CAD) is a major global health issue, with an age-standardized prevalence rate of 3,610.2 per 100,000 people. Furthermore, it remains the leading cause of global cardiovascular (CV) disease mortality, with an age-standardized rate per 100,000 of 108.8 deaths (1). Percutaneous coronary intervention (PCI) is a widely recognized and established treatment for patients with acute coronary syndrome (ACS) and is performed selectively in patients with chronic coronary syndromes (2). Despite significant advances in interventional procedures and medical therapies, addressing the ongoing occurrence of adverse outcomes after PCI remains an important issue (3). Although traditional CV risk factors and various risk scores are currently used to target biological pathways and predict clinical outcomes, this approach does not comprehensively encompass the occurrence of future CV events (4). Cardiac biomarkers and non-invasive cardiac imaging tools play valuable roles in risk stratification and the prediction of future CV events. However, there are several concerns associated with these methods, including their cost, time requirements, potential side effects, and limited predictive power.

In recent years, interest in vascular function measurement in the CV field has increased. Arterial stiffness is a progressive vascular aging process that can be worsened by prolonged exposure to various noxious stimuli such as high blood pressure, hyperglycemia, inflammation, and oxidative stress (5). The measured arterial stiffness index has been significantly associated with the occurrence of future CV events not only in the general population but also in patients with various diseases, even after adjusting for traditional CV risk factors (6–12). Pulse wave velocity (PWV) is a reliable indicator of arterial stiffness and can be obtained by dividing the distance between two different arterial points by the difference in the arrival time of the waveform (13). Numerous studies have identified its incremental value in CV risk assessment. However, the current robust and expanding body of evidence primarily stems from studies conducted in the general population with limited information available on whether these individuals have established CV disease (9, 14, 15). In patients with significant CAD, the prognostic implication of PWV have recently been reported in several studies (7, 16–18), but most of them have limitations regarding sample size, duration of follow-up, and reliability of their results (7, 19). This real-world registry study aimed to investigate the potential association between elevated arterial stiffness measured using brachial-ankle PWV (baPWV) and long-term clinical outcomes in patients who underwent PCI.

## 2 Patients and methods

### 2.1 Study population

**FIGURE 1:**
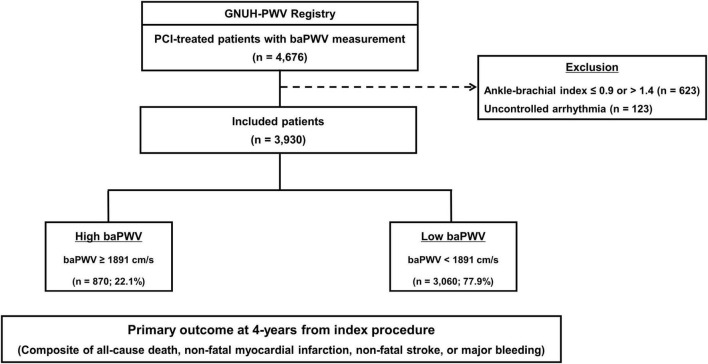
Flow diagram of the study. baPWV, brachial-ankle pulse wave velocity; PCI, percutaneous coronary intervention.

The study population was selected from the multicenter prospective Gyeongsang National University Hospital (G-NUH) registry (NCT04650529) (20). Consecutive patients with significant CAD who underwent PCI (Jinju and Changwon) between January 2010 and November 2018 were enrolled in this registry, which evaluated multiple vascular, hemostatic, and physiological parameters, if available. In the current analysis, we included patients who underwent baPWV measurement during index hospitalization. Of the 4,676 eligible patients, 746 with an ankle-brachial index ≤ 0.9 or >1.4 or uncontrolled arrhythmia because of insufficient accuracy. Finally, 3,930 participants were analyzed (Figure 1).

### 2.2 Patient management and procedures

The patients were treated according to standard practices at the respective hospitals. The choice of treatment strategy (stent implantation and medication) was left to the discretion of the operator based on guidelines (21, 22). Baseline demographic characteristics, CV risk factors, angiographic and procedural parameters, discharge medications, and clinical outcome data were collected through patient interviews or a review of medical records under the supervision of the principal investigator. Further information was collected via telephone, if necessary.

### 2.3 Brachial–ankle pulse wave velocity measurement

The baPWV measurement method has been described previously (6, 23). Briefly, the patient rested in a quiet, temperature-controlled room for at least 5 min before the examination. baPWV was measured using a commercially available volume plethysmographic device (VP-1000; Colin Co., Ltd., Komake, Japan). Pneumatic cuffs were applied to the brachial arteries and ankles bilaterally, electrocardiographic electrodes were placed on both wrists, and phonocardiographic electrodes were placed on the edge of the sternum. The baPWV was calculated by dividing the brachial-ankle distance by the transit time. Well-trained technicians blinded to the protocol evaluated the baPWV based on the approved protocol. In this study, we used the average baPWV values from the left and right measurements.

### 2.4 Study endpoint and definitions

The primary outcome was the occurrence of net adverse clinical events (NACE) within a 4-year period, characterized as a composite of all-cause death, non-fatal myocardial infarction (MI), non-fatal stroke, or major bleeding. Secondary outcomes included major adverse cardiac and cerebrovascular events (MACCE) and major bleeding, both of which were evaluated for up to 4 years. MACCE were defined as a composite of all-cause death, non-fatal myocardial infarction, or non-fatal stroke. Furthermore, major bleeding was specifically defined as Bleeding Academic Research Consortium (BARC) grade 3 or 5 bleeding (24). The other secondary outcomes included each MACCE component.

Clinical outcomes were defined according to the recommendations of the Academic Research Consortium (25). Non-fatal MI was defined as the recurrence of symptoms with the presence of electrocardiographic changes or imaging evidence of new loss of viable myocardium or new regional wall motion abnormalities in associated with an increase in cardiac biomarker levels above the upper limit of normal. Peri-procedural MI was not included as a clinical outcome. Stroke was defined as a neurological deficit attributed to an acute focal injury of the central nervous system by a vascular cause requiring hospitalization and was confirmed by a neurologist based on imaging results. All clinical events were evaluated by an independent event adjudication committee. After discharge from the index hospitalization, patients were routinely followed up at 1, 6, and 12 months after the index procedure and annually thereafter.

### 2.5 Statistical analysis

All categorical variables are presented as numbers and percentages, while continuous variables are presented as the means with standard deviations or medians with interquartile ranges (IQRs) depending on their distribution, which was assessed using the Kolmogorov–Smirnov test. Categorical variables were compared using the chi-squared test or Fisher’s exact test, whereas continuous variables were compared using Student’s t test or Mann–Whitney test.

To determine the optimal cut-off value of baPWV for predicting the primary outcome, we used a time-dependent receiver operating characteristic (ROC) curve analysis. Time-dependent ROC curve analysis was performed using sensitivity and 1 - specificity, which were obtained from various cut-off values of baPWV at 4 years. Youden’s index was used to calculate the optimal cut-off value. Additionally, we classified baPWV into three categories based on a previous study for sensitivity analysis: <1400, ≥1400 & <1800, and ≥1800 cm/s (26).

The associations between baPWV as a continuous variable and the risk of clinical outcomes were fitted using a restricted cubic spline curve with five knots. Cumulative event rates were estimated with the Kaplan–Meier curves and compared using the log-rank test. Hazard ratios (HRs) and 95% confidence intervals (CIs) for primary and secondary outcomes based on the baPWV group were calculated using a Cox proportional hazard regression model. Multivariable Cox proportional hazard models were constructed considering clinically relevant variables such as age, sex, body mass index (BMI), heart rate, mean arterial pressure (systolic blood pressure × 1/3 + diastolic blood pressure × 2/3), presentation with ACS, cigarette smoking, diabetes mellitus, hypertension, dyslipidemia, chronic kidney disease, and the use of potent P2Y_12_ inhibitors, angiotensin blockades, beta-blockers, calcium channel blockers, and statins. The reduced models were constructed through a backward elimination procedure using Akaike’s information criterion. Furthermore, subgroup analysis was performed using multivariable Cox proportional hazard models with specifications including age (≤65, >65 years), sex, ACS status, medical history of hypertension and diabetes mellitus, initial estimated glomerular filtration rate (eGFR, <60 or ≥60 mL/min/1.73 m^2^), and left ventricular ejection fraction (LVEF, <50 or ≥50%). The proportional hazards assumption for the variables in the model was assessed using scaled Schoenfeld residuals. Variable inflation factors (VIFs) were calculated for the variables in the multivariable models, and all the VIFs were below 2, indicating that multicollinearity was not a concern.

All the statistical analyses were conducted using the open-source statistical software R (version 4.2.3)^1^ and R-studio (version 2023.03.1)^2^ and the statistical packages, rms, descr, survival, tableone, survminer, ggplot2, timeROC, and plotRCS. All tests were two-tailed, and a *p* value of <0.05 was considered significant.

## 3 Results

### 3.1 baPWV and baseline characteristics

**FIGURE 2:**
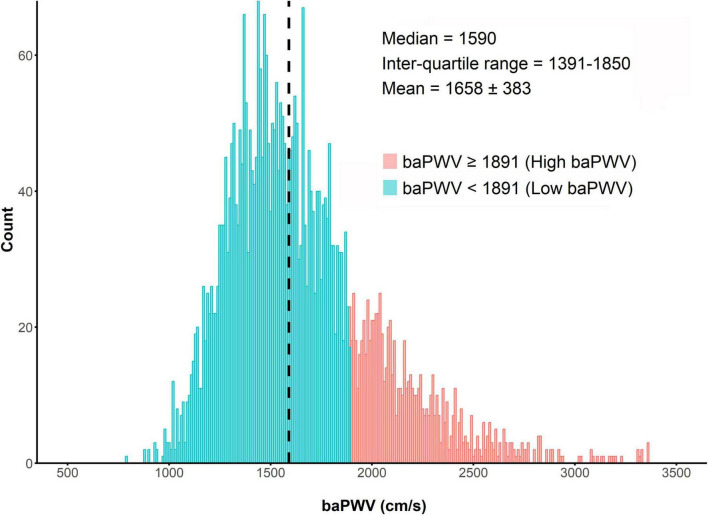
Distribution of baPWV. baPWV, brachial-ankle pulse wave velocity.

The distribution of baPWV values among the enrolled patients (*n* = 3,930) is shown in Figure 2. The median baPWV was 1590 cm/s (intertertile range: 1391, 1850). Supplementary Figure 1 shows the optimal cut-off for baPWV in predicting the primary outcome, which was identified as baPWV = 1891 cm/s. Patients were categorized into two groups according to the baPWV phenotype: 1) the high baPWV group (baPWV ≥ 1891 cm/s; *n* = 870); and 2) the low baPWV group (baPWV < 1891 cm/s; *n* = 3,060).

The baseline characteristics of the enrolled patients according to baPWV are presented in Table 1. The overall age was 64.0 ± 11.5 years, and approximately 70% were men. Approximately 60% of the cohort initially presented with ACS, and most of the patients were treated with drug-eluting stents. The high baPWV group was more likely to be older and have a greater prevalence of female sex and CV comorbidities (e.g., diabetes mellitus, hypertension, chronic kidney disease, and previous stroke) than the low baPWV group, whereas the low baPWV group was more likely to have a greater incidence of cigarette smoking and dyslipidemia than the high baPWV group. Prescriptions for concomitant medications were mostly similar between the groups.

**TABLE 1:** Baseline characteristics according to baPWV.

	baPWV ≥ 1891 cm/s (*n* = 870)	baPWV < 1891 cm/s (*n* = 3,060)	*p*-value
Age, years	71.5 ± 9.9	61.9 ± 11.0	<0.001
Women, *n* (%)	420 (48.3)	734 (24.0)	<0.001
Body mass index, kg/m^2^	23.5 ± 3.1	24.8 ± 3.3	<0.001
Systolic blood pressure, mmHg	133.3 ± 25.3	128.7 ± 23.2	<0.001
Diastolic blood pressure, mmHg	78.4 ± 14.3	78.1 ± 13.7	0.482
Heart rate, /min	77.2 ± 16.0	74.1 ± 15.4	<0.001
Index presentation with ACS, *n* (%)	542 (62.3)	1866 (61.0)	0.506
**Risk factors, n (%)**			
Cigarette smoking	156 (17.9)	1099 (35.9)	<0.001
Diabetes mellitus	379 (43.6)	781 (25.5)	<0.001
Hypertension	592 (68.0)	1384 (45.2)	<0.001
Dyslipidemia	443 (50.9)	1753 (57.3)	0.001
Chronic kidney disease	221 (25.4)	273 (8.9)	<0.001
Previous PCI	138 (15.9)	437 (14.3)	0.267
Previous stroke	70 (8.0)	149 (4.9)	<0.001
**Laboratory findings**			
LVEF, %	55.2 ± 9.6	57.1 ± 8.3	<0.001
eGFR, mL/min/1.73 m^2^	75.7 ± 29.7	88.1 ± 26.2	<0.001
Total cholesterol, mg/dL	174.6 ± 47.3	182.5 ± 47.6	<0.001
LDL cholesterol, mg/dL	110.7 ± 41.6	118.5 ± 42.4	<0.001
HDL cholesterol, mg/dL	46.7 ± 14.4	44.4 ± 12.5	<0.001
Triglyceride, mg/dL	150.1 ± 121.5	166.7 ± 133.3	0.001^a^
Hb_*A*1*c*_, %	6.6 ± 1.3	6.4 ± 1.3	<0.001^a^
**Procedural characteristics**			
AHA/ACC lesion: type B2/C	762 (87.6)	2718 (88.8)	0.342
Multivessel disease, *n* (%)	170 (19.5)	522 (17.1)	0.100
Target lesion, *n* (%)			<0.001
–Left main coronary artery	12 (1.4)	72 (2.4)	
–Left anterior descending artery	487 (56.0)	1704 (55.7)	
–Left circumflex artery	248 (28.5)	762 (24.9)	
–Right coronary artery	297 (34.1)	1076 (35.2)	
Treatment method, *n* (%)			<0.001
–Drug-eluting stent	779 (89.5)	2719 (88.9)	
–Bioresorbable scaffold	2 (0.2)	62 (2.0)	
–Bare metal stent	5 (0.6)	16 (0.5)	
–Drug-coated balloon	31 (3.6)	96 (3.1)	
–POBA	53 (6.1)	167 (5.5)	
Numbers of stent, n	1.5 ± 0.8	1.4 ± 0.7	0.076
Total stent length, mm	38.2 ± 22.4	36.7 ± 21.6	0.092
Stent diameter, mm	3.1 ± 0.5	3.2 ± 0.5	<0.001
**Concomitant medications, n (%)**			
Aspirin	862 (99.1)	3032 (99.1)	1.000
Type of P2Y_12_ inhibitor			0.250
–Clopidogrel	709 (81.5)	2403 (78.5)	
–Prasugrel	34 (3.9)	150 (4.9)	
–Ticagrelor	110 (12.6)	449 (14.7)	
Beta blocker	537 (61.7)	1895 (61.9)	0.944
Angiotensin blockade	609 (70.0)	2159 (70.6)	0.784
Calcium channel blocker	86 (9.9)	217 (7.1)	0.008
Statin	811 (93.2)	2916 (95.3)	0.019

The data are presented as n (%). ACC, American College of Cardiology; ACS, acute coronary syndrome; AHA, American Heart Association; baPWV, brachial-ankle pulse wave velocity; eGFR, estimated glomerular filtration rate; HDL, high-density lipoprotein; LDL, low-density lipoprotein; LVEF, left ventricular ejection fraction; PCI, percutaneous coronary intervention; POBA, plain old balloon angioplasty.

^a^Assessed by nonparametric test.

### 3.2 baPWV and clinical outcomes

**FIGURE 3:**
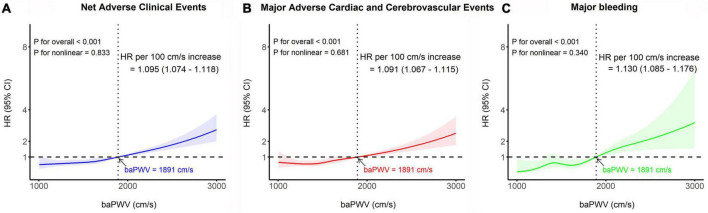
Restricted cubic spline analyses depicting the continuous association between baPWV and the risk of panel **(A)** NACE, **(B)** MACCE, and **(C)** major bleeding. The solid line represents the relative hazard in relation to the cut-off value of baPWV (1891 cm/s), with the shaded area indicating the 95% confidence intervals. baPWV, brachial-ankle pulse wave velocity; CI, confidence interval; HR, hazard ratio; MACCE, major adverse cardiac and cerebrovascular event; NACE, net adverse clinical event.

**FIGURE 4:**
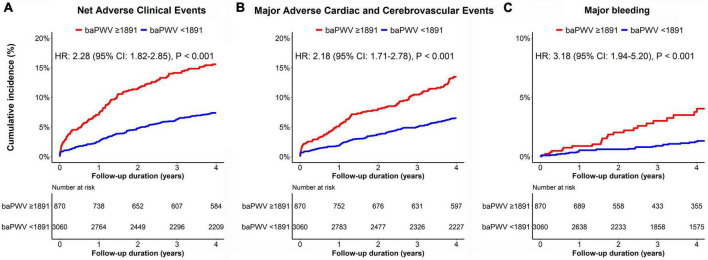
Kaplan–Meier curves for the cumulative incidence of panel **(A)** NACE, **(B)** MACCE, and **(C)** major bleeding according to the baPWV groups. The red line represents clinical events from patients with high baPWV (≥1891 cm/s), while the blue line represents clinical events from subjects with low baPWV (<1891 cm/s). baPWV, brachial-ankle pulse wave velocity; MACCE, major adverse cardiac and cerebrovascular event; NACE, net adverse clinical event.

The median follow-up duration was 3.72 years (IQR: 1.56–6.47 years). During the follow-up period of up to 4 years, 323 (8.2%) NACE occurred in all patients. baPWV was linearly correlated with the risk of clinical events after PCI. baPWV was significantly associated with the risk of NACE (HR per 100 cm/s increase, 1.095; 95% CI, 1.074–1.118; *p* < 0.001), MACCE (HR per 100 cm/s increase, 1.091; 95% CI, 1.067–1.115; *p* < 0.001), and major bleeding (HR per 100 cm/s increase, 1.130; 95% CI, 1.085–1.176; *p* < 0.001) (Figure 3). When comparing clinical outcomes according to the baPWV cut-off value, patients with high baPWV had greater rates of NACE (unadjusted HR: 2.28; 95% CI: 1.82–2.85; *p* < 0.001), MACCE (unadjusted HR: 2.18; 95% CI: 1.71–2.78; *p* < 0.001), and major bleeding (unadjusted HR: 3.18; 95% CI: 1.94–5.20; *p* < 0.001) at the 4-year follow-up than did those with low baPWV (Figure 4).

After adjusting for clinically relevant variables, patients with high baPWV consistently had a greater risk of clinical events including NACE (adjusted HR 1.44; 95% CI,1.12–1.85; *p* = 0.004), MACCE (adjusted HR 1.40; 95% CI, 1.07–83; *p* = 0.015) and major bleeding (adjusted HR 1.94; 95% CI, 1.15–3.25; *p* = 0.012) than did those with low baPWV. The baPWV was also independently associated with the risk of clinical event occurrence as a continuous variable (per 1-SD increase) (Table 2). In the sensitivity analysis with baPWV classified into three categories, we observed a trend of higher risk of NACE with increasing baPWV categories; however, this did not reach statistical significance (Supplementary Table 1).

**TABLE 2:** Incidence and risk of clinical outcomes according to baPWV.

	Cumulative incidence	Crude HR (95% CI)	*p*-value	Adjusted HR^a^ (95% CI)	*p*-value
**NACE^b^ (MACCE^c^ + major bleeding^d^)**					
baPWV (per 1-SD increase)		1.42 (1.31–1.53)	<0.001	1.22 (1.11–1.35)	<0.001
baPWV < 1891 cm/s	200 (6.5)	reference		reference	
baPWV ≥ 1891 cm/s	123 (14.1)	2.28 (1.82–2.85)	<0.001	1.44 (1.12–1.85)	0.004
**MACCE (all-cause death, MI, stroke)**					
baPWV (per 1-SD increase)		1.39 (1.28–1.52)	<0.001	1.19 (1.07–1.33)	0.002
baPWV < 1891 cm/s	174 (5.7)	reference		reference	
baPWV ≥ 1891 cm/s	104 (12.0)	2.18 (1.71–2.78)	<0.001	1.40 (1.07–1.83)	0.015
**Major bleeding**					
baPWV (per 1-SD increase)		1.60 (1.37–1.86)	<0.001	1.44 (1.19–1.74)	<0.001
baPWV < 1891 cm/s	37 (1.2)	reference		reference	
baPWV ≥ 1891 cm/s	28 (3.2)	3.18 (1.94–5.20)	<0.001	1.98 (1.17–3.33)	0.010
**All-cause death**					
baPWV (per 1-SD increase)		1.50 (1.33–1.68)	<0.001	1.24 (1.07–1.45)	0.006
baPWV < 1891 cm/s	68 (2.2)	reference		reference	
baPWV ≥ 1891 cm/s	52 (6.0)	2.75 (1.92–3.94)	<0.001	1.48 (0.99–2.19)	0.055
**Non-fatal MI**					
baPWV (per 1-SD increase)		1.23 (1.04–1.45)	0.015	1.24 (1.05–1.47)	0.012
baPWV < 1891 cm/s	58 (1.9)	reference		reference	
baPWV ≥ 1891 cm/s	34 (3.9)	2.10 (1.37–3.20)	0.001	1.62 (1.03–2.53)	0.035
**Non-fatal Stroke**					
baPWV (per 1-SD increase)		1.37 (1.14–1.64)	0.001	1.08 (0.84–1.38)	0.555
baPWV < 1891 cm/s	36 (1.2)	reference		reference	
baPWV ≥ 1891 cm/s	25 (2.9)	2.48 (1.49–4.14)	<0.001	1.42 (0.81–2.50)	0.223

baPWV, brachial-ankle pulse wave velocity; CI, confidence interval; HR, hazard ratio; MACCE, major adverse cardiac and cerebrovascular events; MI, myocardial infarction; NACE, net adverse clinical events.

^a^Multivariable analysis was performed adjusting clinically relevant covariates, including age, sex, body mass index, heart rate, mean arterial pressure, presented with acute coronary syndrome, diabetes mellitus, hypertension, dyslipidemia, chronic kidney disease, cigarette smoking, potent P2Y_12_ inhibitor, angiotensin blockade, beta blocker, calcium channel blocker, and statin.

^b^NACE were defined as a composite of all-cause death, non-fatal myocardial infarction, non-fatal stroke, or major bleeding.

^c^MACCE were defined as a composite of all-cause death, non-fatal myocardial infarction, or non-fatal stroke.

^d^Major bleeding was defined as Bleeding Academic Research Consortium (BARC) 3 or 5 bleeding.

**FIGURE 5:**
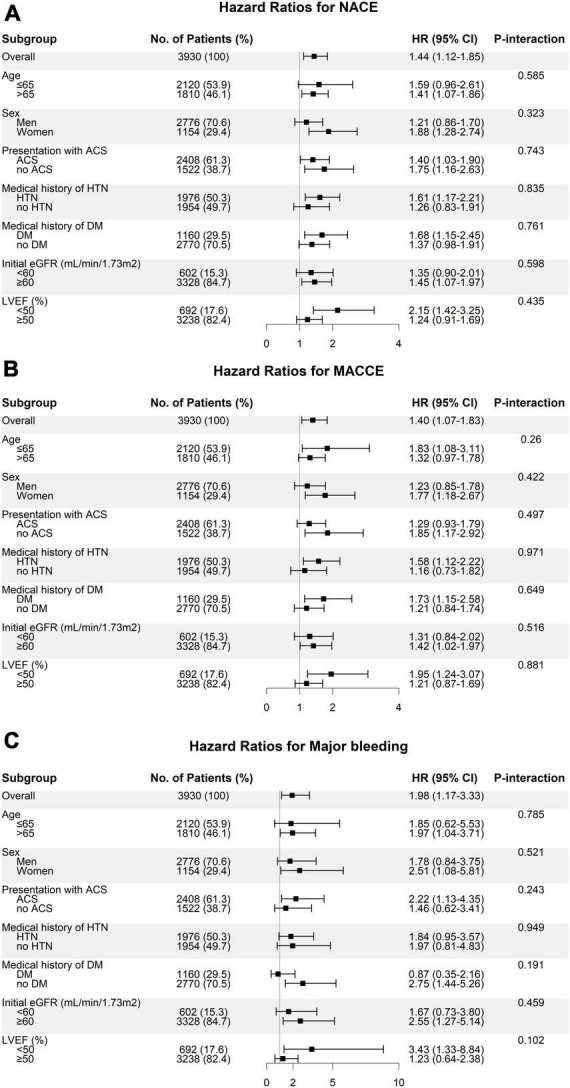
Subgroup analysis of the risk of panel **(A)** NACE, **(B)** MACCE, and **(C)** major bleeding according to the baPWV groups. Hazard ratios represent the risk of clinical outcomes in patients with high baPWV (≥1891 cm/s) compared to subjects with low baPWV (<1891 cm/s). ACS, acute coronary syndrome; baPWV, brachial-ankle pulse wave velocity; DM, diabetes mellitus; eGFR, estimated glomerular filtration rate; HTN, hypertension; LVEF, left ventricular ejection fraction; MACCE, major adverse cardiac and cerebrovascular event; NACE, net adverse clinical event.

We evaluated the clinical impact of high baPWV on the occurrence of clinical events across subgroups (Figure 5). Compared to patients with baPWV values lower than the cutoff, patients with baPWV values higher than the cutoff showed a consistently worse prognosis in terms of NACE, MACCE, and major bleeding across all subgroups, with no significant interactions. In particular, clinical impact of high vs. low baPWV phenotype on NACE occurrence was consistently significant, regardless of age (>65 vs. ≤65 years old) (Supplementary Figure 2).

## 4 Discussion

To the best of our knowledge, the present study is the largest to evaluate the long-term association between the arterial stiffness index and clinical outcomes in high-risk patients with CAD. This is the first demonstration of a close link between baPWV and ischemic and bleeding events in patients who underwent PCI. The key findings were as follows: (1) the 4-year risks of ischemic and bleeding events increased with increasing baPWV values; (2) the optimal cutoff value of baPWV for predicting future clinical events was 1891 cm/s; and (3) patients with baPWV values higher than the cutoffs consistently exhibited a greater risk for clinical outcomes including NACE, MACCE, and major bleeding, even after adjusting for clinically relevant covariates.

Arterial stiffness serves as an early marker of functional and structural changes in the arterial walls. Extensive research has established that elevated arterial stiffness is an independent risk factor for atherosclerotic CV disease and has prognostic value beyond conventional risk factors. This association is observed not only in the general population but also in patients with various CV conditions (6–9).

Recent studies have highlighted an association between arterial stiffness and coronary atherosclerosis. However, these studies primarily demonstrated the correlation between the presence/severity of CAD and baPWV (7, 16, 27, 28). There is limited evidence regarding the long-term prognostic implications of baPWV in these populations (29–33). The current study provides the largest-sized clinical evidence to date demonstrating a link between baseline baPWV and long-term clinical outcomes in high-risk patients with CAD, specifically those who underwent PCI. Notably, this analysis also revealed a novel finding by establishing a significant relationship between baseline baPWV and the occurrence of major bleeding, in addition to ischemic events.

Several hypotheses have been proposed to explain the mechanisms underlying these findings. First, arterial stiffness may reflect the cumulative deleterious effects of CV risk factors on the arterial wall over a long period of time. Previous studies have consistently reported that PWV is closely influenced by major risk factors for CV events, including abnormal lipid phenotype, insulin resistance, and blood pressure. Our study also showed that patients with high PWV had more traditional CV risk factors and comorbidities than those with low PWV (26, 34, 35). Second, arterial stiffness is associated with endothelial dysfunction, platelet activation, and plaque vulnerability; the latter are significantly associated with the development and prognosis of CAD (36, 37). Third, arterial stiffness may influence the hemodynamic indices of coronary-vascular system that are associated with poor prognosis in patients with CAD. For example, arterial stiffness is positively associated with myocardial wall stress and negatively associated with coronary perfusion and coronary flow reserve (38–40). Finally, arterial stiffness may reflect the degree of vascular fragility (41, 42). Endothelial cells play a role in all major hemostatic pathways following vascular injury, limiting clot formation to areas where hemostasis is needed to restore vascular integrity. Although dual antiplatelet therapy is essential for reducing the risk of ischemic events after PCI, its potency can also increase the risk of bleeding. Therefore, an increase in arterial stiffness may be considered an indicator of vascular vulnerability related to risk of bleeding during antithrombotic therapy.

This study has several important clinical implications. First, PWV has been widely recognized in arterial hypertension guidelines as an indicator of target organ damage and a predictor of CV events (14, 43). Our findings provide valuable evidence for extending the clinical application of PWV to other disease groups, particularly those with high-risk profile such as CAD. Second, despite a significant reduction in the incidence of recurrent events after PCI compared to that in the past, it is still notably high (44). As identifying these high-risk groups remains essential, baPWV may be a promising marker for identifying patients at an elevated risk of recurrent events following successful PCI. The improved identification of high-risk patients will enable better risk stratification and more effective preventive therapies. In high-risk patients with elevated PWV, PWV can serve as a target or monitoring tool for interventions aimed at lowering CV risk. Pharmacological approaches such as antihypertensive drugs, statins, and nitrates, as well as non-pharmacological strategies (e.g., weight reduction, exercise, reduced salt intake, and moderate alcohol consumption), may offer potential benefits in managing this high-risk phenotype (13). However, further studies are needed to confirm whether reducing the PWV using these approaches directly improves the clinical prognosis of patients with CV disease. Further research is required to explore the prognostic utility of a PWV-guided approach for secondary prevention in patients with CAD who have undergone PCI.

This study has several limitations. First, the data were derived from a prospective, two-center observational registry. Despite rigorous adjustments for known risk factors, there were potentially unmeasured confounding factors. Second, although approximately 4,000 patients were consecutively included in the analysis, there was a possibility of selection bias because we excluded relatively common comorbidities such as peripheral artery disease and arrhythmia, which could affect the accuracy of baPWV. However, this study included a large sample size with a comparable representation of all-comers and prospective data collection by well-trained coordinators. Third, important variables such as antiplatelet agent compliance and post-PCI smoking cessation were not available, which could have influenced the observed outcomes. Fourth, the use of various medications, such as antiplatelet agents, statins, and antihypertensive agents, during or after PCI may have influenced the baPWV measurements. Finally, baPWV values were based on baseline measurements, and follow-up baPWV values were not considered.

## 5 Conclusion

The arterial stiffness index, as assessed using baPWV, was significantly associated with long-term adverse clinical outcomes, including both ischemic and bleeding events, in patients with CAD who underwent PCI. These findings suggest that baPWV measurements may serve as a valuable tool for risk stratification and prognostic assessment in high-risk patients with CAD, providing important clinical implications for the management and prevention of future CV events. Further research is warranted to explore the potential benefits of interventions that target arterial stiffness in this patient population.

## Data availability statement

The datasets used in this study are not publicly available due to restrictions related to hospital-based data. Requests for access to the datasets should be directed to Y-HJ, goodoctor@naver.com.

## Ethics statement

The studies involving humans were approved by the respective hospitals of institutional registry. The studies were conducted in accordance with the local legislation and institutional requirements. The ethics committee/institutional review board waived the requirement of written informed consent for participation from the participants or the participants’ legal guardians/next of kin because this study used anonymized institutional registry data (NCT04650529).

## Author contributions

BK: Writing – original draft, Data curation, Formal analysis. J-HS: Writing – original draft, Methodology, Formal analysis, Visualization. J-HA: Data curation, Writing – review and editing. MK: Data curation, Validation, Writing – review and editing. K-HK: Data curation, Writing – review and editing. JB: Methodology, Writing – review and editing. YC: Data curation, Writing – review and editing. J-SK: Data curation, Writing – review and editing. YP: Data curation, Writing – review and editing. S-JH: Data curation, Writing – review and editing. UT: Data curation, Writing – review and editing. PG: Data curation, Writing – review and editing. J-YH: Conceptualization, Writing – review and editing. Y-HJ: Conceptualization, Writing – review and editing, Supervision.

## Footnotes

1
www.R-project.org

2
www.rstudio.com
