# Supplementary material for: Long-term prognostic implications of brachial-ankle pulse wave velocity in patients undergoing percutaneous coronary intervention
Source: Front Med (Lausanne). 2024 Jun 7;11:1384981. doi: 10.3389/fmed.2024.1384981 (PMC11190319; doi:10.3389/fmed.2024.1384981)
Supplement: Supplementary file 1 [file Data_Sheet_1.docx]

Supplementary Material

Long-Term Prognostic Implications of Brachial-Ankle Pulse Wave Velocity in Patients Undergoing Percutaneous Coronary Intervention

Byung Sik Kim^†^, Jong-Hwa Ahn^†^, Jeong-Hun Shin^*^, Min Gyu Kang, Kye-Hwan Kim, Jae Seok Bae, Yun Ho Cho, Jin-Sin Koh, Yongwhi Park, Seok-Jae Hwang, Udaya S. Tantry, Paul A. Gurbel, Jin-Yong Hwang, Young-Hoon Jeong^*^

*** Correspondence:** Corresponding Author

Jeong-Hun Shin, MD, PhD
[cardio.hyapex@gmail.com](mailto:cardio.hyapex@gmail.com)

Young-Hoon Jeong, MD, PhD
[goodoctor@naver.com](mailto:goodoctor@naver.com)

# Supplementary Figures and Tables

## Supplementary Figures


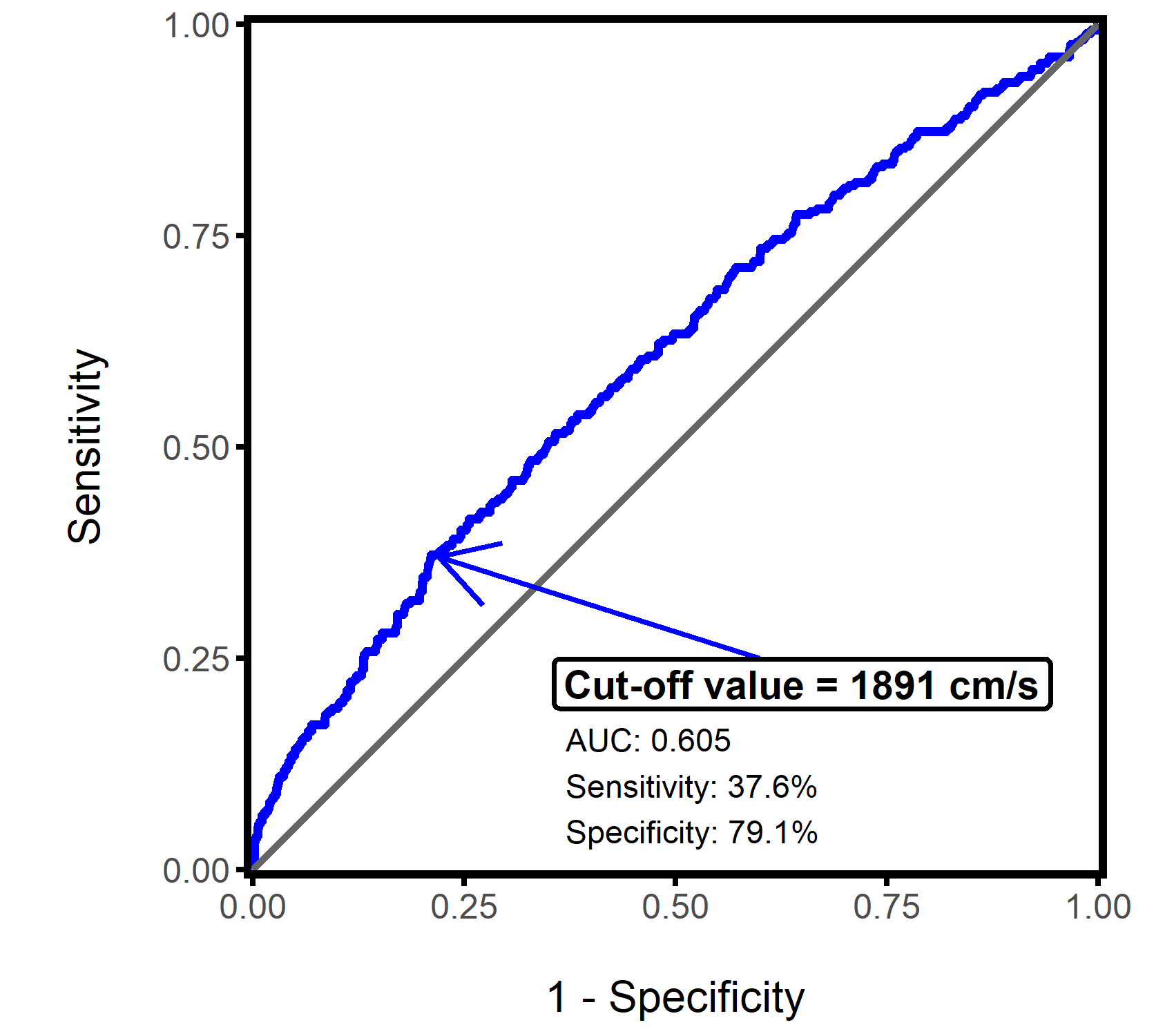


**Supplementary Figure 1.** Optimal cut-off for baPWV in predicting the primary outcome

baPWV, brachial-ankle pulse wave velocity; AUC, area under the curve.


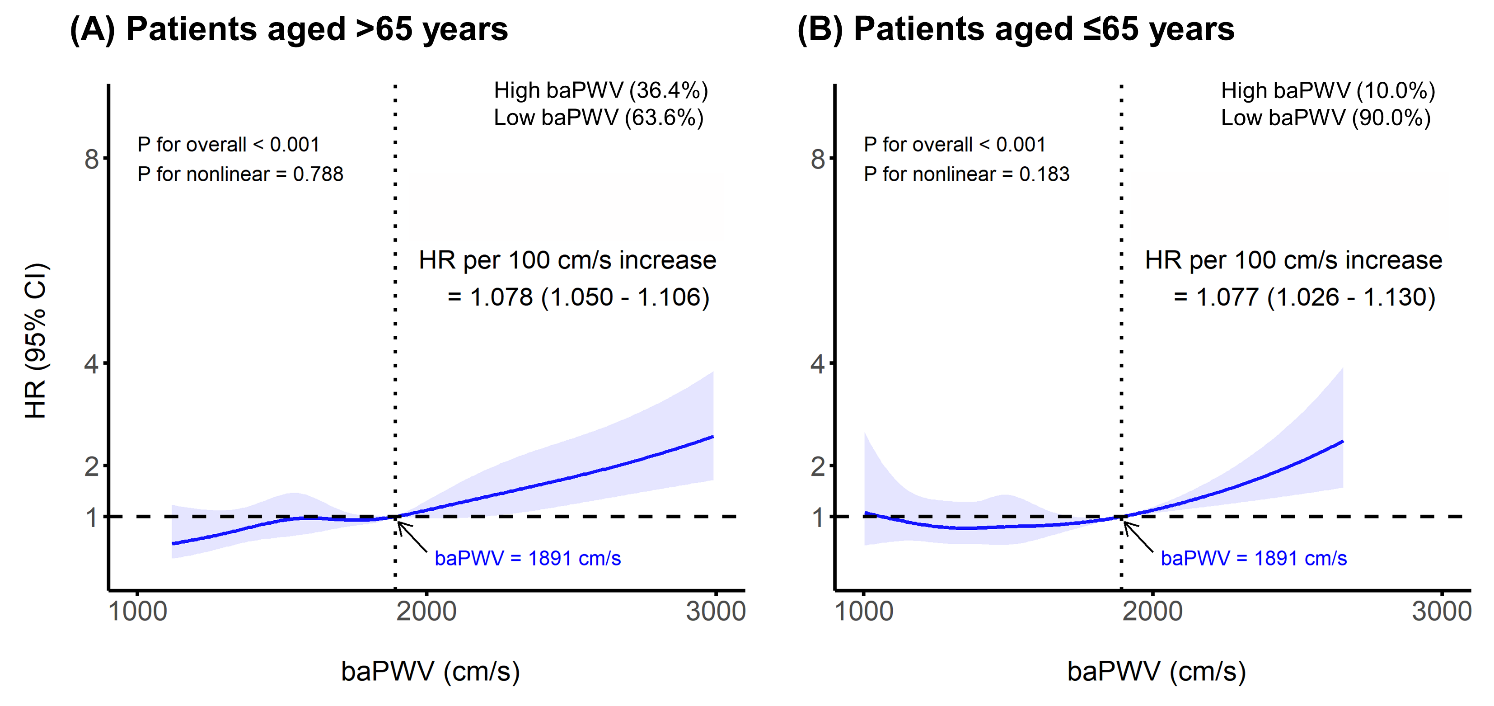


**Supplementary Figure 2.** Continuous association between baPWV and the risk of NACE according to age (A) >65 years old (B) ≤65 years old

baPWV, brachial-ankle pulse wave velocity; CI, confidence interval; HR, hazard ratio; NACE, net adverse clinical event.

## Supplementary Table

| **Supplementary Table. Incidence and risk of clinical outcomes according to baPWV category (****<1400, ≥1400 & <1800, ≥1800 cm/s)** | | | | | | |  |
| --- | --- | --- | --- | --- | --- | --- | --- |
|  | Cumulative incidence | Crude HR  (95% CI) | *p* value | Adjusted HR^a^  (95% CI) | *p* value | *p* value for trend | |
| **NACE**^b^ **(MACCE**^c^ **+ major bleeding**^d^**)** |  |  |  |  |  | 0.039 | |
| baPWV <1400 cm/s | 57 (5.6) | reference |  | reference |  |  | |
| baPWV ≥1400 & <1800 cm/s | 127 (7.1) | 1.28 (0.94–1.75) | 0.121 | 1.01 (0.73–1.40) | 0.939 |  | |
| baPWV ≥1800 cm/s | 139 (12.4) | 2.33 (1.71–3.17) | <0.001 | 1.39 (0.98–1.97) | 0.068 |  | |
| **MACCE (all-cause death, MI, stroke)** |  |  |  |  |  | 0.150 | |
| baPWV <1400 cm/s | 49 (4.8) | reference |  | reference |  |  | |
| baPWV ≥1400 & <1800 cm/s | 111 (6.2) | 1.30 (0.93–1.81) | 0.131 | 1.00 (0.71–1.42) | 0.980 |  | |
| baPWV ≥1800 cm/s | 118 (10.5) | 2.27 (1.63–3.17) | <0.001 | 1.30 (0.89–1.90) | 0.171 |  | |
| **Major bleeding** |  |  |  |  |  | 0.019 | |
| baPWV <1400 cm/s | 9 (0.9) | reference |  | reference |  |  | |
| baPWV ≥1400 & <1800 cm/s | 25 (1.4) | 1.70 (0.79–3.63) | 0.174 | 1.60 (0.74–3.44) | 0.230 |  | |
| baPWV ≥1800 cm/s | 31 (2.8) | 3.88 (1.85–8.17) | <0.001 | 2.75 (1.28–5.90) | 0.010 |  | |
| **All-cause death** |  |  |  |  |  | 0.019 | |
| baPWV <1400 cm/s | 15 (1.5) | reference |  | reference |  |  | |
| baPWV ≥1400 & <1800 cm/s | 43 (2.4) | 1.64 (0.91–2.95) | 0.099 | 1.19 (0.65–2.17) | 0.580 |  | |
| baPWV ≥1800 cm/s | 62 (5.5) | 3.86 (2.20–6.79) | <0.001 | 1.98 (1.07–3.66) | 0.030 |  | |
| **Non-fatal MI** |  |  |  |  |  | 0.324 | |
| baPWV <1400 cm/s | 21 (2.1) | reference |  | reference |  |  | |
| baPWV ≥1400 & <1800 cm/s | 36 (2.0) | 0.98 (0.57–1.67) | 0.932 | 0.91 (0.53–1.57) | 0.742 |  | |
| baPWV ≥1800 cm/s | 35 (3.1) | 1.54 (0.90–2.65) | 0.117 | 1.30 (0.75–2.27) | 0.354 |  | |
| **Non-fatal Stroke** |  |  |  |  |  | 0.551 | |
| baPWV <1400 cm/s | 10 (1.0) | reference |  | reference |  |  | |
| baPWV ≥1400 & <1800 cm/s | 23 (1.3) | 1.31 (0.63–2.76) | 0.473 | 0.95 (0.45–2.04) | 0.901 |  | |
| baPWV ≥1800 cm/s | 28 (2.5) | 2.59 (1.26–5.34) | 0.010 | 1.30 (0.59–2.89) | 0.515 |  | |

baPWV, brachial-ankle pulse wave velocity; CI, confidence interval; HR, hazard ratio; MACCE, major adverse cardiac and cerebrovascular events; MI, myocardial infarction; NACE, net adverse clinical events.

^a^ Multivariable analysis was performed adjusting clinically relevant covariates, including age, sex, body mass index, heart rate, mean arterial pressure, presented with acute coronary syndrome, diabetes mellitus, hypertension, dyslipidemia, chronic kidney disease, cigarette smoking, potent P2Y_12_ inhibitor, angiotensin blockade, beta blocker, calcium channel blocker, and statin.

^b^ NACE were defined as a composite of all-cause death, non-fatal myocardial infarction, non-fatal stroke, or major bleeding.

^c^ MACCE were defined as a composite of all-cause death, non-fatal myocardial infarction, or non-fatal stroke.

^d^ Major bleeding was defined as Bleeding Academic Research Consortium (BARC) 3 or 5 bleeding.
